# The impact of distance education on the socialization of college students in the Covid-19 era: problems in communication and impact on mental health

**DOI:** 10.1186/s12909-024-05551-7

**Published:** 2024-05-24

**Authors:** Qingxia Liu, Douxiu Lin

**Affiliations:** 1 Mental Health Education Counseling Center, Student Affairs Office, Anhui Sanlian University, Hefei, China; 2 Department of Education, School of Culture and Media, Anhui Xinhua University, Hefei, China

**Keywords:** COVID-19, Higher education students, Loneliness, Online learning, Psychological well-being, Self-directed learning, Social anxiety, Social emotional learning, Social interaction

## Abstract

**Background:**

The problems of students’ social interaction and psychological well-being associated with online learning dependent on self-directed learning have become an important topic of research in recent years worldwide due to the COVID-19 pandemic, affecting their Social Emotional Learning. This paper aimed to compare the students’ loneliness, social anxiety, social interaction, and general psychological well-being at different stages of online learning (at the beginning and the height of the pandemic), considering their criteria (presence/absence of a job and own family).

**Methods:**

For this, the researchers conducted an electronic survey of students (*n* = 320) twice, in February and May 2020, using four questionnaires: UCLA loneliness scale-3, Social Anxiety Scale for E-Learning Environments, Social Interaction Scale, and Brief Adjustment Scale. The responses at different stages of online learning were compared using Student’s t-test. Differences between employed and unemployed students with or without their own families were determined using the analysis of variance (ANOVA).

**Results:**

The findings showed that unemployed students without their families suffered the most from loneliness. Social interaction online was rated higher by students with their own families; psychological well-being at the beginning of the distance period and social anxiety at the height of the distance period were higher among unemployed students.

**Conclusions:**

This research can become a theoretical basis for a phase-by-phase study of social predictors for the psychological well-being of higher education students and is of practical value for teachers and administrators of online learning aimed at students’ socialization. In addition, it provides education officials with information about how students perceive psychological well-being, anxiety, social interaction, and loneliness during distance learning, which can help officials direct their decisions and reforms to improve interaction in the online environment.

## Background

According to the Sustainable Development Goals (SDG), education is a driving force for progress, a common good, and an inalienable human right [1, 2]. The coronavirus infection (COVID-19) is an acute, sometimes severe, respiratory illness caused by a novel coronavirus, SARS-CoV-2 [3]. The spread of COVID-19 infection has led to a pandemic that has affected the education sector [4]. Bans for travel and face-to-face education catalyzed online interaction [1, 2]. According to UNESCO statistics, 91% of students were forced to switch to distance learning [5]. These data indicate the scale of the impact caused by the COVID-19 pandemic on the educational process. The transition to distance learning was due to quarantine measures introduced in different countries to prevent the spread of the virus.

Distance learning (online learning, e-learning) is a form of education where the teacher and students are physically separated and use different technologies to interact in the educational process in teacher-student and student-student formats [6]. During distance learning, students may face new challenges that affect their self-discovery. Therefore, distance learning makes students deal with the issue of self-discovery. Self-discovery is the process of comprehending and understanding one’s own self, inner beliefs, values, and purpose in life [7, 8]. Changes in the mode and learning environment can force students to rethink their learning focus and goals. The lack of direct communication with teachers and classmates can increase internal dialogue and self-reflection. Distance learning may require students to be more independent, helping them identify their strengths and weaknesses.

Self-directed learning (SDL) is a process in which a person takes the initiative (with or without the help of others) and attempts to understand: what the learning needs are, how to formulate the goal of their learning, how to identify human and material resources for learning, how to choose and implement a learning strategy, and how to evaluate the learning outcomes [7]. The introduction of new online solutions, tools, and applications has revolutionized global education [8]; self-directed learning models have allowed some students to improve individual performance, time management, and self-regulation skills [9]. The closure of schools and colleges as a result of quarantine measures greatly limited the ability of students to maintain communication and cooperation with peers and teachers [10]. According to previous studies [7–9], the introduction of self-directed learning and new online tools in global education has marked a transition from traditional forms of learning to more flexible, individualized, and targeted approaches to education. This transition can have a positive impact on the learning process and student outcomes.

The issues of students’ social interaction and mental well-being in distance learning became a prominent research topic in the subsequent pandemic stages [11–13]. This type of social interaction and mental well-being is called Social Emotional Learning (SEL). It combines the processes by which knowledge, skills, and attitudes are acquired and applied to develop a healthy personality, the ability to interact, empathize, care for others, take responsibility for decisions, achieve individual and collective goals, and manage emotions [14]. Both SDL and SEL are recognized as “21st Century Skills” [15, 16]. Compared to 2019 (pre-pandemic period), students in 2021 (pandemic period) self-directing their learning experienced a lag in their socio-emotional development, according to a survey of school district leaders. Among teachers surveyed, 33% mentioned student socio-emotional health as the second most important distance learning challenge after academic achievement [14]. The results of the study indicate the need to pay attention to the social and emotional health of students during the transition to distance learning. In this period, students especially require full psychological and social support. The COVID-19 pandemic thus represents a natural experiment in which socialization is limited as a learning factor during imposed SDL [17].

According to Joosten and Cusatis [18], socialization is a measure of preference or need for social interaction and communication with peers and the teacher. Social interaction is a communicative (verbal, non-verbal) interaction between two or more sides of the educational process [19]. The researchers consider socialization in the educational process not as a secondary process but as an important variable that determines motivation and learning outcomes; moreover, it has an impact on success in future professional activities [20]. The opposite of socialization is social isolation [19]. Social anxiety and loneliness are among the first reactions to social isolation [19]. Subjective perception of shortcomings in socialization breeds loneliness [21]. It is a psychological experience that can be painful and have consequences for students’ mental health and well-being [21–23]. Thus, the results of the reviewed papers [19–21] show that social isolation can have serious consequences for the mental health and well-being of students. It is important to pay attention to psychological assistance and social integration to support students and their well-being.

Peer communication is important to students’ mental health and well-being [6, 24]. Loneliness becomes an essential aspect of students’ psychological state, especially during distance learning. Separation from the community of classmates and teachers can lead to a sense of social isolation. Loneliness can affect the mental health of students, causing stress, anxiety, and low mood. However, loneliness can also be a chance for personal growth, reflection, and focus on personal interests. Research confirms the importance of communication among college students: a quarter of Turkish students surveyed mentioned loneliness and lack of opportunity to get together as a result of COVID-19 quarantine restrictions as the most critical barrier in their lives [25]. An increase in cyberbullying cases has also been reported in response to student loneliness and psychological distress [26]. These results highlight the need to develop strategies to support students in colleges during periods of social isolation. In particular, effective measures can be opportunities for virtual social interaction and psychological support.

Loneliness and self-discovery are issues associated with distance learning. The feeling of loneliness can increase due to distance from the traditional resources of the student environment. In turn, a decrease in personal communication can deepen the sense of distance and influence the formation of social connections. As a result, students can find themselves in a state of structural uncertainty, which creates additional difficulties for self-discovery and adaptation to new conditions [6, 25, 27]. These aspects suggest the importance of researching the relationship between self-discovery, loneliness, and the psychological state of students during distance learning. In a study conducted in the United States, the use of Twitter by school districts throughout the country during the early COVID-19 pandemic was examined [27]. The results showed that communication using Twitter focused its messaging on one of three primary purposes: broadcasting announcements, building community, and conducting routine school business unrelated to the pandemic. The predominant mode of interaction was confirmation or collaboration in the message exchange, demonstrating the importance of such online communication in maintaining students’ well-being. When studying remotely, students have the opportunity to communicate in a digital format. However, a limiting factor in this message exchange is that students may not see each other’s honest face-to-face reactions, as the camera and microphone can be turned off at any time [4, 28].

The researchers have different opinions as to the effectiveness of communication in digital format using smartphones, social networks, websites, and thematic forums [28]. It has been reported that, in distance courses, the students noted a lack of both connectedness and a sense of community [29], while according to other data, distance courses helped to develop connectedness and a sense of community [12], especially where efforts were made to provide emotional support [30]. Hehir et al. [24] noted that participants of distance education have different needs for communication and team sense. In addition, college and university students may have other relationships where communication and connectedness are a priority for them (work, family). Therefore, the autonomy of distance education for them may be preferable to live communication of full-time learning. Thus, the studies suggest individual differences between students and their needs in interaction and communication in the period of distance education. It is crucial to consider these aspects when developing and implementing distance learning programs.

In the USA, the Evidence Project [31] provides comprehensive reports on the COVID-19 pandemic’s impact on students, according to which a significant proportion (30–40%) experience mental and socio-emotional health problems. Thus, students, who studied remotely for a long time, became marginalized and more often experienced negative effects. Anxiety and suicide attempts, which rose before the pandemic, rose even more at the beginning of the pandemic, especially among females [13]. Despite good results or even improvements in academic outcomes, these positive effects did not last long, as virtual absenteeism tends to increase with each new stage of distance education [14]. Therefore, it is important to explore not a one-time study of the transition to distance learning, when the new opportunities that have opened up give a positive initial impression, but different stages of distance learning.

The transition of the education system from a traditional to an online context during COVID-19 required consideration of several factors to ensure student satisfaction with e-learning. One Malaysian study aimed to investigate the factors influencing student satisfaction with e-learning during the COVID-19 crisis [32]. Data were collected using a questionnaire that assessed four factors influencing student satisfaction with e-learning during the COVID-19 crisis (i.e., instructor performance, course evaluation, student factors, and system quality) and were analyzed using partial least squares-structural equation modeling (PLS-SEM). The results indicated that four factors were significantly associated with student satisfaction with e-learning during COVID-19. Student factors and system quality were the major factors determining student satisfaction with e-learning. The findings point to a statistically significant relationship between instructor performance, student factors, course evaluation, and system quality in student satisfaction. Additionally, the results demonstrate that both course evaluation and system quality consistently mediate the relationship between instructor performance and student satisfaction.

Another study aimed to examine the impact of the COVID-19 pandemic on psychological consequences (depression, anxiety, and insomnia) [33]. According to the results, participants with post-traumatic stress disorder (*n* = 360) demonstrated a higher level of depression, anxiety, and insomnia compared to participants without post-traumatic stress disorder (*n* = 639). The results highlight the need to pay attention to psychological well-being and provide support for individuals with psychological problems, especially in the face of pandemics and stress.

Analyzing the abovementioned literature, it can be observed that the topic of the influence of distance education on students’ socialization in the era of COVID-19 remains relevant today. The educational system’s transition from a traditional to an online context poses a challenge. Existing research has some gaps: studies indicate limited opportunities for digital interaction, especially when cameras and microphones are inactive. In this case, it may be challenging to observe honest reactions from other participants [4, 28]. Among researchers, there is uncertainty and disagreement about the effectiveness of digital communication via smartphones, social media, and other online means [28, 29]. This paper gives new insights into the impact of online learning on student social interaction and psychological well-being during the COVID-19 pandemic. Using a multi-factor approach, the study compares loneliness, social anxiety, social interaction, and psychological well-being at different stages of online learning. The authors consider such criteria as job availability/absence and family. The study provides a dynamic analysis of psychological well-being and social interaction at the beginning and height of the pandemic. The analysis demonstrates changes over time and determines the stages with the most significant impact on students. The article focuses on the context of distance learning, studying its specifics and impact on social interaction and psychological well-being.

### Problem statement

The digital revolution in education has generated desocialization for the educational process participants [20]. In this regard, loneliness and social anxiety are of concern as some principal issues of social isolation that affect the individual’s mental health [34]. It is equally important to study social interaction, that is, communication with peers and a teacher within self-directed distance education [22]. It should be taken into account that students are a diverse social group: some of them work at jobs, in parallel with their studies, and some already have their own families. These differences can significantly impact the level of loneliness or social anxiety.

It is important to compare the characteristics of loneliness, social anxiety, social interaction, and general psychological well-being in different phases of distance education to trace the possible dynamics, for example, at the beginning of distance learning and at its height [22]. It is also important to study the overall psychological well-being of online students and to establish whether there are differences at different stages of distance learning. This is of significant importance as indicators of anxiety, loneliness, and social well-being have an impact on the overall state of students, their productivity, motivation, learning, work dynamics, and other important factors [11, 33, 35].

The purpose of this work is to compare the social predictors of students’ psychological problems at different stages of the self-directed distance learning imposed during the COVID-19 pandemic in China: in February 2020, when distance learning had just begun as a result of the pandemic, and when it had already developed and lost its initial novelty, in May 2020. The study spanned from February to May, encompassing four months. This will include consideration of the individual characteristics of students, their employment, and the presence of their own families.

## Materials and methods

### Study design

A two-way analysis of variance (ANOVA) was conducted to determine differences between employed and unemployed students. ANOVA was chosen by analogy with the study by Halpern et al. [36], which examined the influence of students’ criteria on the answers to questionnaires on loneliness and personal and social well-being. In the context of COVID-19, ANOVA has also been employed, for example, in the study by Jahangiry et al. [37]. The analysis of variance was utilized to investigate how people perceive the outbreak of COVID-19.

### Participants

The study was conducted at Anhui Sanlian University, Hefei, China, from February 2020 to May 2020 and enrolled 320 students (152 males and 168 females). Table 1 shows their demographic characteristics. The main selection criterion was exclusive distance learning from February to May 2020. In addition, the students about whom the faculty administration had data as employed or with their own families received personal electronic invitations to participate in the study; the rest of the students took part after seeing information about the study on the university website. Having their own family meant having a husband/wife and one or more children. Thus, the selection criteria include both objective (exclusive distance learning) and subjective (the presence of a job or family) aspects to create a diverse and representative group of participants.

**Table 1:** Demographics of the study participants

	Females	Males	Age
Employed	66	94	20–35
Unemployed	102	58	18–21
With own family	29	22	25–35
Without own family	139	130	18–24

### Research procedure

The study considers two phases of distance education:

- Phase (1) Beginning of distance education, 2–3 weeks after the introduction of quarantine measures and the transition to online (February 2020).
- Phase (2) The height of distance education and full-scale studies (May 2020).

### Data collection and analysis

The researchers used Google Forms for data collection. All participants were of legal age and provided written consent. No personal information was collected. Through Google Forms, the participants accessed the survey and completed it in a convenient place (home, cafe, etc.) and from any device connected to the Internet. The participants provided responses without disclosing their identity - this approach encouraged honest and unbiased feedback.

The survey response rates of students were encoded in the SPSS program by assigning the highest score of 1 (5/5 = 1) “very good,” 0.8 (4/3 = 0.8) points “good”, etc. The students’ responses in each of the phases were compared using the Student’s t-test. It helped identify any statistically significant changes in the responses over time. A two-way analysis of variance (ANOVA) was conducted to determine the differences between employed and unemployed students with and without their own families.

#### Loneliness

The authors employed the University of California’s third version of the UCLA loneliness scale to evaluate loneliness. The third version is more simplified than the previous ones, where the readability is improved, and the answers’ format and the items’ wording are simplified [38]. It has proven to be a reliable tool in a sample of college students [22]. All 20 items of the questionnaire begin with the wording: “How often do you feel …?” Each item was rated from 1 (never) to 4 (always) on a Likert scale.

#### Social anxiety

To assess social anxiety experienced by students in the online learning environment, Social Anxiety Scale for E-Learning Environments (SASE) was used, where the items were rated from 1 (strongly disagree) to 7 points (strongly agree). SASE was designed to evaluate student-student and teacher-student interactions. The selection of the SASE scale was based on its ease of use. The analysis suggests that SASE is a dependable and credible measurement instrument that effectively evaluates the degree of social anxiety in learners engaged in online education [39, 40].

#### Social interaction

To assess social interaction during distance learning, the authors of this study developed their tool, Social Interaction Scale (SIS). It was based on questions related to social interaction used by previous researchers [10, 18]. The items were rated from 1 (never) to 4 (always).

The researchers invited four experts (two in learning technologies, one in clinical psychology, and one in social psychology) to review each item of the developed SIS test and the items on the remaining questionnaires for validity. Based on their comments, some items were removed as repetitive, and some wordings were simplified.

#### Psychological well-being

The psychological well-being of students was assessed using the Brief Adjustment Scale, BASE-6 [41]: self-reports on a 7-point scale from 1 (not at all) to 7 (extremely). This scale was chosen for its ease of use.

### Ethical principles

This study adheres to important ethical principles that are the standard for scientific research. Below are some key ethical aspects:

**Description of** procedures: all students received information about the purpose of the study and its procedures. **The consent of the participants**: participants participated voluntarily after receiving personal invitations or viewing information on the university’s website. **Demographic data**: Table 1 shows only the demographic characteristics of participants, without specifying their personal identification data. **Personal information protection**: students who took part in the study were employed or had families – this information was not discussed between the participants. Therefore, no one knew whether a particular participant had a family or a job. **Experimental conditions**: the study included the introduction of a new educational course – the participants received detailed instructions and participated in conditions that met ethical standards.

## Results

### Loneliness

Table 2 shows the results of the UCLA loneliness scale. The first column contains the question number, and the second the wording after “How often do you feel …”. Columns 3–4 show the sample mean of students’ assessments for the two phases: at the beginning of a transition to distance education (Phase 1) and during the distance education (Phase 2).

**Table 2:** Student’s t-test results for the UCLA Loneliness scale

		Phase 1	Phase 2	*p*_value_
Q1	Harmony with fellow students	2.73	1.69	0.023*
Q2	Lack of communication	3.14	2.95	0.041*
Q3	A feeling of communication fatigue	1.22	2.36	0.062*
Q4	Difficulty in finding common topics of conversation	1.95	1.97	0.891
Q5	Group membership	2.96	2.80	0.456
Q6	Commonality of interest	2.85	1.94	0.001*
Q7	No sense of closeness	3.15	3.18	0.605
Q8	Spontaneity and immediacy in communication	2.01	2.69	0.069*
Q9	Good mood in communication with fellow students	1.67	1.60	0.784
Q10	Friendliness	3.02	3.14	0.512
Q11	Feeling deprived of communication	2.65	2.89	0.031*
Q12	A feeling of importance in relationships with fellow students	2.2	1.89	0.042*
Q13	Nobody understands you	2.21	2.19	0.625
Q14	Isolation in the team	2.32	2.86	0.026*
Q15	Ability to find company whenever you want	3.11	3.04	0.698
Q16	Feeling that there are people who understand you	2.76	2.29	0.001*
Q17	Feeling shy	2.32	3.12	0.125
Q18	Feeling lonely	2.54	2.19	0.218
Q19	There are people you are happy to talk to	3.12	2.44	0.001*
Q20	There are people, who will support and help you	2.96	2.81	0.321

* - Significant difference (*p* < 0.05)

Student’s t-test (*p* < 0.05) showed statistically significant differences in student responses between Phase 1 and Phase 2 for 10 items of the questionnaire. Students report a lack of communication, an increase in feelings of isolation, misunderstanding, and a decrease in common interests among them. At the same time, there is statistically significant progress in spontaneity and immediacy in communication, which can be explained by the selection of more suitable distance education tools, which made it possible to add some spontaneity to the routine educational process. Despite the negative mood that prevailed in both phases, the survey participants still had fellow students, who supported them, there were topics for conversation, and the sense of belonging had not been lost.

### Social interaction

Table 3 shows the results for SIS. Statistically significant differences between the two phases are observed in interaction with colleagues and teachers in a distance format (in Phase 1, an interaction was rated at an average level, while in Phase 2, the ratings were lower), in the suitability of online learning tools (on the height of distance learning, respondents found them less suitable than at the beginning), as well as in difficulties in online learning (their number was lower in Phase 2).

**Table 3:** Student’s t-test results for the SIS

		Phase 1	Phase 2	*p*_value_
Q1	You are satisfied with your interaction with colleagues and the teacher during distance learning	2.30	1.59	0.026*
Q2	You find the tools available good enough for interaction	2.92	1.76	0.049*
Q3	The distance format is better for interaction	2.45	2.22	0.79
Q4	You enjoy working in a team online	2.89	2.55	0.27
Q5	You still have difficulties during team working in a distance format	2.61	2.12	0.036*
Q6	You find it difficult to complete collective tasks online	2.59	2.94	0.64

* - Significant difference (*p* < 0.05)

### Social anxiety

Table 4 shows the results for SASE. T-test did not reveal statistically significant differences in social anxiety between different phases of the distance education period. The respondents rated all items close to the average level. Socialization, defined as the preference for social interaction with peers and instructors, significantly influences students’ perception, anxiety, and satisfaction with their learning experience [18].

**Table 4:** Student’s t-test results for the SASE

		Phase 1	Phase 2	*p*_value_
Q1	In online learning, I am afraid of being misunderstood when communicating with a teacher	3.22	2.90	0.73
Q2	In online learning, I am afraid of being criticized by fellow students	1.13	1.36	0.65
Q3	I feel uncomfortable in e-learning discussions	2.96	3.15	0.62
Q4	I feel embarrassed when communicating with an online teacher	3.04	3.10	0.81
Q5	In online learning, I avoid asking fellow students questions	3.15	3.19	0.76
Q6	I prefer to remain silent when I need to interact with an e-learning instructor	3.22	3.06	0.70

* - Significant difference (*p* < 0.05)

### Psychological well-being

The final questionnaire concerned the psychological well-being of the respondents. It does not carry information about the influence of the distance learning period on students but informs about their general psychological adaptation. Table 5 shows the results for BASE-6.

**Table 5:** Student’s t-test results for the BASE-6

		Phase 1	Phase 2	*p*_value_
Q1	To what extent have you felt irritable/resentful?	2.22	2.3	0.53
Q2	To what extent have you felt anxious/afraid?	3.13	3.06	0.45
Q3	To what extent have you felt unhappy?	2.96	3.15	0.22
Q4	How much has emotional stress interfered with feeling good about yourself this week?	3.04	3.1	0.77
Q5	How much has emotional stress interfered with your relationships this week?	2.15	4.19	0.03*
Q6	How much has emotional stress interfered with your ability to study this week?	2.22	3.06	0.02*

* - Significant difference (*p* < 0.05)

Statistically significant differences are observed in the last two questions: emotional stress interfered with relationships with others and the learning process more in Phase 2 than in Phase 1. Students did not record an increase in irritability and anxiety and did not feel more unhappy at the height of the distance period compared to the beginning; the overall level of emotional stress also did not change.

Table 6 shows the results of ANOVA.

**Table 6:** ANOVA results

	Loneliness		Social Interaction		Social Anxiety		Psychological Well-Being	
	Phase 1	Phase 2	Phase 1	Phase 2	Phase 1	Phase 2	Phase 1	Phase 2
Sample mean	2.54	2.50	2.63	2.20	2.79	2.79	2.62	3.14
Employed	2.33*	2.39*	2.58	2.21	2.67	2.51*	2.24*	3.11
Unemployed	2.78*	2.7*	2.65	2.18	2.89	3.07*	2.99*	3.17
With own family	2.22*	2.31*	2.84*	2.31*	2.91	2.68	2.51	3.21
Without own family	2.86*	2.69*	2.42*	2.08*	2.65	2.90	2.73	3.06

* - Significant difference (*p* < 0.05)

A two-way analysis of variance (ANOVA) (*p* < 0.05) confirmed a statistically significant difference between the answers of employed and unemployed students for loneliness in both phases, social anxiety in Phase 2, and psychological well-being in Phase 1. The statistically significant difference between students with and without their families is observed for loneliness and social interaction in both phases.

## Discussion

Interaction between people determines thoughts, moods, behaviours, and feelings and can enhance positive emotions and psychological well-being [22]. Social isolation and the transition to SDL in the online mode have dramatically changed the lives of university students [42]. Previous studies have shown that loneliness exacerbates depression and adjustment problems [22] and provokes aggressive and addictive [43, 44]. At the same time, belonging to a team and a college and psychological comfort among fellow students soften loneliness and serve as a protective barrier in violation of psychological health and well-being [22]. The sense of belonging – a subjective feeling of deep connection to social groups, physical places, as well as individual and collective experiences – is a fundamental human need that predicts numerous psychological, physical, social, economic, and behavioural outcomes [45]. It is possible that students may feel a sense of belonging (for example, because the material they are studying is inherently interesting) but may not feel part of a team – thus lacking a sense of belonging to the team. As these findings showed, students who switched to SDL in distance learning had a rather high level of loneliness both at the beginning and at the height of distance learning and generally low psychological well-being. They rated their social interaction higher at the beginning of the distance period (Table 6: sample mean = 2.63). In Phase 2, it was also above the average (sample mean = 2.20). The high rates of social interaction are easily explained by Adnan and Anwar [4]: well-chosen digital interventions help increase team cohesion and facilitate collaborative work on projects. At the same time, interaction within study assignments was insufficient to overcome the overall loneliness.

Authors from Indonesia [42] reported on the general psychological well-being of students during the pandemic at a slightly above-average level. However, in this research, neither at the beginning nor during online studies did students record even an average level of psychological well-being as the scores were low (Table 6: 2.62 in Phase 1 and 3.14 in Phase 2 out of the maximum possible 7 points). Among Chinese students’ earlier reports indicated that during the initial stages of the pandemic, certain publications referred to the Chinese population as potential carriers of SARS-CoV-2.1 and as responsible for causing the pandemic [26]. Such reports had the potential to negatively impact the psychological well-being of Chinese students.

The purpose of the article by Canadian scientists was to study the impact of the COVID-19 pandemic on the mental health of first-year students [46]. The research focus was *student well-being and academic success at U-Flourish Queens University*. As part of the study, three consecutive cohorts of undergraduate students enrolled in 2018 (before the pandemic), 2019 (transition period), and 2020 (during the pandemic) completed electronic surveys at the beginning and at the end of their first year of study. Clinically significant symptoms of depression, anxiety, insomnia, and self-harm were more commonly reported in the 2020–2021 cohort, which coincided with distance learning and pandemic restrictions. In this study, employed students were less anxious at the first stage. In Phase 2, this advantage increased even more. The anxiety of students decreased only in Phase 2: in general, they managed to adapt to the new environment.

Another factor that could have negatively influenced students’ psychological well-being was the isolation they experienced as a result of the pandemic, which was not well understood at the time. As a consequence, students may have had a lower level of psychological well-being unnecessarily due to engaging in the SDL necessary for distance. If it weren’t for COVID-19, they might not have encountered difficulties with SDL and remote education. The fact that COVID-19 can be a primary cause for the decline in students’ psychological well-being is evidenced by the increasing distress students experienced by May 2020. They endured the impacts of COVID-19 for a longer duration than initially anticipated (Table 5).

According to the author from Cyprus, Demetriou [47], employed students from Cyprus experienced higher anxiety compared to unemployed ones. In this paper, on the contrary, employed students were less anxious in Phase 1. In Phase 2, this advantage increased even more (Table 6: 2.67 for employed vs. 2.89 for unemployed in Phase 1 and 2.51 for employed vs. 3.07 for unemployed in Phase 2). Differences in anxiety among Chinese students can be explained by the popularity of the same electronic communication tools in school and at work [4]. Employed students were already familiar with the communication and interaction tools used because they used them for communication and interaction at work (e-mail, forums, and online conferences). In the past few years, even before the pandemic, office workers used such tools very often. As a result, employed students tend to be more comfortable with SDL in distance learning. The study among Cypriot students concluded that the more time students spent online, the more stressed they were [47]. According to the current research findings, students’ anxiety decreased in Phase 2: Chinese students generally adapted to the new environments.

An author from Asia [10] has studied social interaction issues in the context of online learning during the pandemic. The results showed that social interaction has a significant positive impact on the effectiveness of online learning. This study revealed statistically significant differences between the two phases in interaction with colleagues and teachers in a distance format (in Phase 1, interaction was rated at an average level, while in Phase 2, the ratings were lower), in the suitability of online learning tools (in the midst of distance learning, respondents considered them less suitable than at the beginning), and in difficulties associated with online learning (their number was lower in Phase 2).

**Fig. 1:**
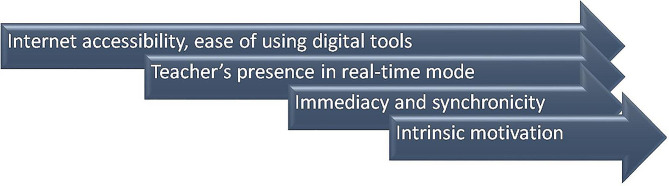
Keys to effective online communication and interaction, according to Hehir et al. [24]

British scientists [24] suggest several keys to the effectiveness of remote communication and solving the problem of isolation (Fig. 1). The first key is the availability and stability of the Internet and digital tools for distance learning and the convenience of their use. The second key is the teacher’s presence in real-time mode when they present the material, answer questions here and now, and direct the vectors of students’ work. The third key is immediacy and synchronicity, which is achieved through instant feedback between teacher and student, student and student. The fourth key is intrinsic motivation. The current study has some similarities with the above findings of the previous research: not all students equally need and are motivated or interested in socializing with their peers. This conclusion is supported by the current findings of differences between employed and unemployed students with and without families.

A study by authors from the United States showed how mental health and well-being associated with COVID differed between undergraduate and graduate students [48]. According to the results, undergraduate students reported a higher perception of stress, more repetitive negative thoughts, less positive attitudes, and less support from faculty than graduate students. In the current study, employed students were less anxious at the first stage. In Phase 2, this advantage increased even more: 2.67 for the employed versus 2.89 for the unemployed in Phase 1 and 2.51 for the employed versus 3.07 for the unemployed in Phase 2.

### Research limitations

The study included only one university in China. This study has a rather small sample focused on a single region, which allows conclusions taking into account the Chinese context. In addition, the questionnaires used in the study were slightly modified from the original versions and translated into Chinese. The version of the questionnaires used in the study was approved by the expert group, but a rigorous statistical analysis of validity (including Cronbach’s alpha, Exploratory Factor Analysis, and Confirmatory Factor Analysis) was not carried out.

## Conclusion

This study was aimed at a comparative assessment of students’ SEL with respect to their loneliness, anxiety, social interaction, and general psychological well- being with SDL during online education, considering their employment and having their own families at different stages of the distance period. The general psychological well-being of employed and unemployed students differed only at baseline and was higher in unemployed ones.

This study makes a significant contribution to educational psychology and social sciences for several key reasons. First, it analyzes the impact of distance learning on the psychological state of students, considering its various stages, in particular at the beginning and during a pandemic. At the same time, new data on loneliness, social anxiety, social interaction, and psychological well-being provide a comprehensive view of the psychological state of students. These features make the study unique.

The study can serve as a theoretical basis for further stages of research into social factors that affect the psychological well-being of students in online learning. The results of the study have direct practical value for teachers and administrators of online learning. The presented data can help increase the quality of online education and guide reforms to improve the psychological state of students. The findings of this study will be valuable for policymakers in terms of decision-making and implementing reforms to enhance online interaction. By identifying the key areas of focus in online education, officials will now have a better understanding of which factors require greater attention to make SDL in distance education more comfortable for all participants. The study examines social predictors of the psychological well-being of higher education applicants, opening up opportunities for further details in this area. Thus, the distinguishing features of this research are its relevance, complex topic, and practical significance for further elaborations related to educational psychology.

## Data Availability

The datasets used and/or analysed during the current study are available from the corresponding author on reasonable request.

## Abbreviations

ANOVA: Analysis of variance
PLS-SEM: Partial least squares-structural equation modeling
SASE: Social Anxiety Scale for E-Learning Environments
SDG: Sustainable Development Goals
SDL: Self-directed learning
SEL: Social Emotional Learning
SIS: Social Interaction Scale
